# Upregulation of P2X3 receptors in primary afferent pathways involves in colon-to-bladder cross-sensitization in rats

**DOI:** 10.3389/fphys.2022.920044

**Published:** 2022-09-08

**Authors:** XingYou Dong, Yang Yang, Shengjun Luo, Xiaohong Deng, Wei Tang

**Affiliations:** ^1^ Department of Urology, Chongqing General Hospital, University of Chinese Academy of Sciences, Chongqing, China; ^2^ Department of Urology, People's Hospital of Shapingba District, Chongqing, China; ^3^ Department of Urology, The First Affiliated Hospital of Chongqing Medical University, Chongqing, China

**Keywords:** cross-organ sensitization, colitis, bladder overactivity, P2X3 receptor, TNBS

## Abstract

**Background:** Clinical investigation indicates a high level of co-morbidity between bladder overactivity and irritable bowel syndrome. The cross-sensitization of afferent pathways has been demonstrated to be the main reason for the cross-organ sensitization, but the underlying mechanism is unclear.

**Methods:** A single dose of 2, 4, 6-trinitrobenzene sulfonic acid (TNBS) was applied to induce the colitis rat models by intracolonic administration. All rats were randomly divided into three groups: control, TNBS-3-day, and TNBS-7-day groups. Western blot and immunofluorescent staining were performed to detect the expression of the P2X3 receptor. The spontaneous contractions of the detrusor strip were measured to evaluate the detrusor contractility function. The micturition function was measured by a cystometry experiment. The intercontractile interval (ICI) and maximum bladder pressure (BP) were recorded.

**Results:** The distal colon from colitis showed serious tissue damage or chronic inflammation after TNBS instillation (*p* < 0.01). However, there were no detectable histological changes in bladder among groups (*p* > 0.05). TNBS-induced colitis significantly increased P2X3 receptor expression on the myenteric and submucosal plexus of the distal colon and urothelium of the bladder, especially at day 3 post-TNBS (*p* < 0.05). Meanwhile, the expression of the P2X3 receptor on DRG neurons was increased in TNBS-induced colitis (*p* < 0.01). The detrusor strip of rats exhibited detrusor overactivity after days 3 and 7 of TNBS administration (*p* < 0.01), but inhibition of the P2X3 receptor had no effect (*p* > 0.05). Moreover, the rats with colitis exhibited the micturition pattern of bladder overactivity, manifested by decreased ICI and increased maximum BP (*p* < 0.05). Interestingly, inhibition of the P2X3 receptor by intrathecal injection of A-317491 alleviated bladder overactivity evoked by TNBS-induced colitis (*p* < 0.05).

**Conclusion:** The upregulation of the P2X3 receptor in an afferent pathway involved in bladder overactivity evoked by TNBS-induced colonic inflammation, suggesting that the P2X3 receptor antagonist may be an available and novel strategy for the control of bladder overactivity.

## 1 Introduction

Bladder overactivity (OAB) is the common symptom of bladder functional disease, characterized by increased micturition frequency, urgency, and nocturia (Alagiri et al., 1997; Kim et al., 2020). The mechanism and pathogeny of OAB remain unclear, but increasing clinical epidemiological investigation indicates a high level of co-morbidity between OAB and irritable bowel syndrome (IBS) (Alagiri et al., 1997; Francis et al., 1997; Bullones Rodriguez et al., 2013). Patients with IBS are more likely to experience symptoms of bladder overactivity than the non-IBS population, including nocturia, increased urinary frequency, and urge incontinence (Terruzzi et al., 1992; Cukier et al., 1997; Brumovsky and Gebhart, 2010). In experimental models of colitis, colonic inflammation results in the appearance of neurogenic cystitis, characterized by irritative micturition patterns and an increase in micturition frequency (Pezzone et al., 2005; Ustinova et al., 2006).

The cross-sensitization of primary afferent pathways has been widely recognized as the mechanism underlying cross-organ sensitization (Malykhina, 2007; Ustinova et al., 2010). Specifically, retrograde-tracing studies have revealed that the afferent nerve fibers innervating the bladder and colon are partially overlapped at the level of dorsal root ganglia (DRG) neurons and second-order neurons of the spinal cord (Qin et al., 2005; Christianson et al., 2007; Herrity et al., 2014). In rodent models of colitis induced by intracolonic TNBS administration, colonic irritation enhances the resting firing rates of bladder C-fibers and contributes to mechanical hypersensitivity in response to normal bladder distension (Ustinova et al., 2006; Ustinova et al., 2007). Conversely, cyclophosphamide-induced cystitis in mice also increases the mechanical sensitivity of afferent to colorectal distension and the percentage of chemosensitive afferent (Brumovsky et al., 2009). Furthermore, both distal colon and bladder-innervating DRG neurons exhibit hyperexcitability (Beyak et al., 2004; Malykhina et al., 2006; Lei and Malykhina, 2012). These results suggest that the sensitization of colonic afferent pathways induced by colonic inflammation can result in cross-sensitization of bladder afferent pathways. However, the mechanisms underlying the effect of colonic inflammation on bladder afferent pathways remain unclear.

The P2X3 receptor, a ligand-gated ion channel receptor of extracellular adenosine 5′-triphosphate (ATP), is mainly expressed in primary sensory afferent pathways, especially in the small- and middle-sized DRG neurons (Shinoda et al., 2009). Increasing studies have shown that P2X3 receptor knockout mice display significant attenuation in bladder micturition reflex (Cockayne et al., 2000; Dang et al., 2008). Interestingly, in the patients and animal models of IBS or cystitis, the P2X3 receptor is increased in the afferent pathways of the colon and bladder (Yiangou et al., 2001; Tempest et al., 2004; Dang et al., 2008; Deiteren et al., 2015). Immunohistochemistry showed that the P2X3 receptor was localized to submucosal ganglia and myenteric plexus of the distal colon (Poole et al., 2002; Van Nassauw et al., 2002). The inhibition of P2X3 receptor was demonstrated to reverse visceral hypersensitivity in acute TNBS-induced colitis (Deiteren et al., 2015). Similarly, pharmacological blockade or genetic knockout of the P2X3 receptor alleviates the bladder overactivity in the animal models of cystitis (Ito et al., 2008). However, the underlying mechanism of the P2X3 receptor on colon-to-bladder cross-sensitization remains unclear.

In the present study, the rat model of colitis was established with TNBS by intrarectal administration, and the effect of P2X3 receptors on bladder overactivity evoked by TNBS-induced colonic inflammation was investigated. These results will provide a new effective treatment strategy for colon-to-bladder cross-sensitization.

## 2 Materials and methods

### 2.1 Animals and models

Female Sprague–Dawley (SD) rats (about 250 g) were purchased from the Experimental Animal Center of Army Medical University (Chongqing, China). All rats were housed in cages with a 12 h light/dark cycle and a designated temperature of 25 ± 2 °C. All animals were allowed free access to enough food and water. The phase of the estrous cycle in female rats was determined by vaginal smears, and non-estrous female rats were used for the study to avoid the interference of the estrous cycle. All experimental procedures and protocols were approved by the Institutional Animal Care and Use Committee of Army Medical University (protocol number: AMUWEC20212093) and conformed to the National Institutes of Health Guide for the Care and Use of Laboratory Animals. All rats were randomly divided into three groups: the control group, TNBS-3-day group, and TNBS-7-day group. According to a previous report (Ustinova et al., 2007), a single dose of 2, 4, 6-trinitrobenzene sulfonic acid (TNBS) was applied to induce colitis in rats by intrarectal administration. After being anesthetized under isoflurane, the TNBS-3-day and TNBS-7-day rats were administered an intrarectal enema (8 cm from the anus) of 100 mg/kg TNBS dissolved in 50% ethanol. Control rats were administered an intrarectal saline enema. Morphological assessment of colitis was determined by the disease activity index (DAI, 0–12 score) as previously described (Nooh and Nour-Eldien, 2016; Da Silva et al., 2018) and clinically characterized by the presence of loose stools or diarrhea (0–4 sore), rectal bleeding (0–4 sore), and significant weight loss (0–4 sore).

### 2.2 Tissue sections and staining

After 3 or 7 days of intrarectal administration, the rats (*n* = 4 for each group) were anesthetized with excess sodium pentobarbital. Subsequently, segments of colon and bladder tissues were removed and fixed with 4% paraformaldehyde. After being dehydrated and embedded in paraffin, the colon and bladder tissues were cut into 5-μm-thick tissue sections. The hematoxylin and eosin staining was performed on the colon and bladder tissue sections to observe the pathological change. The thickness of the muscular wall of the distal colon was measured, and the visceral damage score was performed by providing scores ranging from 1 to 5, according to inflammation, infiltration, and vascular density of the tissue. (1, no inflammation; 2, very low inflammation; 3, low level of infiltration; 4, high level of infiltration and vascular density; 5, transmural infiltrations and high vascular density). The increases in the thickness of the muscular wall, the width of the submucosal space (lamina propria), and the depth of the mucosal layer were also considered signs of inflammation.

### 2.3 Western blotting

After being anesthetized with sodium pentobarbital (60 mg/kg, i.p), the bladder and distal colon (*n* = 3 for each group) were removed and homogenized into RIPA lysis buffer (Beyotime, Shanghai, China) on ice for 30 min. Followed by centrifugation at 12,000 g for 10 min, the supernatant was collected, and the protein concentration was measured using a BCA protein assay kit (Bio-Rad, Hercules, United States). Total protein of 30 µg was separated on 10% SDS-PAGE and transferred to nitrocellulose membranes (Merck Millipore, Darmstadt, Germany). After being blocked with 5% non-fat milk for 1 h, the membranes were incubated overnight at 4°C with a mouse anti-P2X3 antibody (1:100, sc-390572, Santa Cruz Biotechnology) or anti-GAPDH antibody (mouse, 1:1000, ab8245, Abcam). The membranes were incubated with the horseradish peroxidase-conjugated secondary antibody (1:5000, A0258, Beyotime, Shanghai, China), followed by washing with TBST three times. Finally, the protein bands were visualized using the ECL substrate (Thermo Fisher, Rockfort, IL). The intensity of the protein band was analyzed using ImageJ and normalized by the intensity of GAPDH.

### 2.4 Immunofluorescence

After being anesthetized with sodium pentobarbital (60 mg/kg, i.p), rats (n = 4 for each group) were transcardially perfused with 0.9% normal saline, followed by 4% paraformaldehyde. Then, the L6-S1 DRG was removed and fixed with 4% paraformaldehyde. After dehydrated overnight with 30% sucrose solution in 4% paraformaldehyde, the DRG tissues were cut into 25-μm-thick tissue sections for immunofluorescent staining. For paraffin sectioning of colon and bladder tissues, the sections were routinely deparaffinized and rehydrated for immunofluorescent staining. Briefly, the sections were washed with 0.01 M PBS three times and blocked with 5% donkey serum for 1 h at room temperature. Subsequently, the sections were incubated overnight at 4°C with a mouse anti-P2X3 antibody (1:100, sc-390572, Santa Cruz Biotechnology), diluted in 1% BSA and 0.3% Triton X-100. The sections were incubated at room temperature for 2 h with the 555 anti-mouse IgG (1:1000, ab150114, Abcam). Subsequently, the 2-(4-amidinophenyl)-6-indolecarbamidine dihydrochloride (DAPI, 1:1000, Beyotime) staining was performed to label cell nuclei, and the sections were mounted with the antiquenching mounting solution. The images of stained sections were captured on a confocal laser scanning microscope (Olympus, Tokyo, Japan) under the same resolution and parameters. ImageJ software (National Institutes of Health) was used to calculate the fluorescent intensity of P2X3 expression with the corrected total cryosection fluorescence (CTCF) published on the website (http://sciencetechblog.com/2011/05/24/measuring-cell- fluorescence-using-imagej). Briefly, settings such as the threshold and area, the integrated density, and the mean gray value were measured in all images. An area of the section untreated with the primary antibody was selected as the background. Finally, the normalized fluorescent intensity was normalized by the average value of the control group.

### 2.5 Contractility study

The whole bladder (*n* = 4 for each group) was removed after being anesthetized with sodium pentobarbital and placed into Kreb’s solution containing the following (in mM): 119 NaCl, 4.7 KCl, 25 NaHCO_3_, 2.5 CaCl_2_, 1.2 MgSO_4_, 1.2 KH_2_PO_4_, and 11 glucose (pH adjusted to 7.4 with NaOH). The contractility experiments of the bladder strip were prepared as described previously (Liu et al., 2018). Briefly, the bladders were longitudinally cut into strips (about 2 × 8 mm in size), and the strips were vertically suspended in a thermostatically controlled organ bath (37°C) containing 10 ml of Kreb’s solution, aerated with a mixture of 95% O_2_ and 5% CO_2_. One end of the strips was fixed at the bottom of the organ bath, while the other end of strips was connected to a tension transducer (JZJ01, Chengdu Instrument Factory, China). The signals of the tension transducer were recorded and analyzed using a multichannel signal processing system (RM6240C, Chengdu Instrument Factory, China). After equilibrating for 30 min, a 0.75 g of tension was applied to strips to evoke spontaneous contractility. After recording stable spontaneous contraction for 10 min, A-317491 was applied to the surface of the strips by bath. The phasic amplitude of spontaneous contractions were analyzed and compared among groups.

### 2.6 Intrathecal injection

The intrathecal injection was performed according to the previously described procedure (Yang et al., 2021). Briefly, the rats (*n* = 4 for each group) were anesthetized intraperitoneally with urethane (1 g/kg), and a dorsal skin incision was made on the surface of L4–L5 lumbar vertebrae. The intervertebral ligament was cautiously dissected to expose the L4–L5 intervertebral space, followed by dissecting the ligaments and muscles. Subsequently, a 20-G needle was stabbed into the subdural space of rats from the L4–L5 intervertebral space. A polyethylene catheter was inserted into the needle, and the tip of the catheter was advanced to the L6–S1 vertebral level. After removing the needle, the incision was sutured, and A-317491 was administrated in a volume of 20 ul through the other tip of the catheter.

### 2.7 Cystometry

The cystometry experiment was performed under urethane (1 g/kg, intraperitoneally) anesthesia as previously described (Andersson, 2015). Then, followed by making a lower midline abdominal incision to the exposed bladder, a polyethylene catheter (PE50, Smiths Medical, United States) was inserted into the dome of the bladder, and the catheter was held in place with a purse-string suture. Subsequently, the other end of the catheter was connected via a three-way valve to a syringe pump (AVI 270, Smiths Medical, Minnesota, United States) for infusing saline into the bladder (10 ml/h), and to a pressure transducer (YPJ01, Chengdu Instrument Factory, China) for recording intravesical pressure. The signals of the pressure transducer were recorded and analyzed using a multichannel signal processing system (RM6240C, Chengdu Instrument Factory, China). After recording stable intravesical pressure for 30 min, 10 μg A-317491 was administrated via intrathecal injection. The intercontractile interval (ICI) and basal and maximum bladder pressure (BP) were analyzed and compared among groups.

### 2.8 Statistical analysis

All data values are expressed as mean ± SEM, and statistical analyses were performed using IBM SPSS version 20. The difference in the damage score of the colon and bladder was measured using the Kruskal–Wallis H-test. Statistical analyses of measurement data were performed using Student’s t-test or one-way ANOVA followed by Newman–Keuls post hoc tests, with *p* < 0.05 considered significant.

## 3 Results

### 3.1 The histological evaluation of the distal colon and bladder in TNBS-induced colitis

**FIGURE 1:**
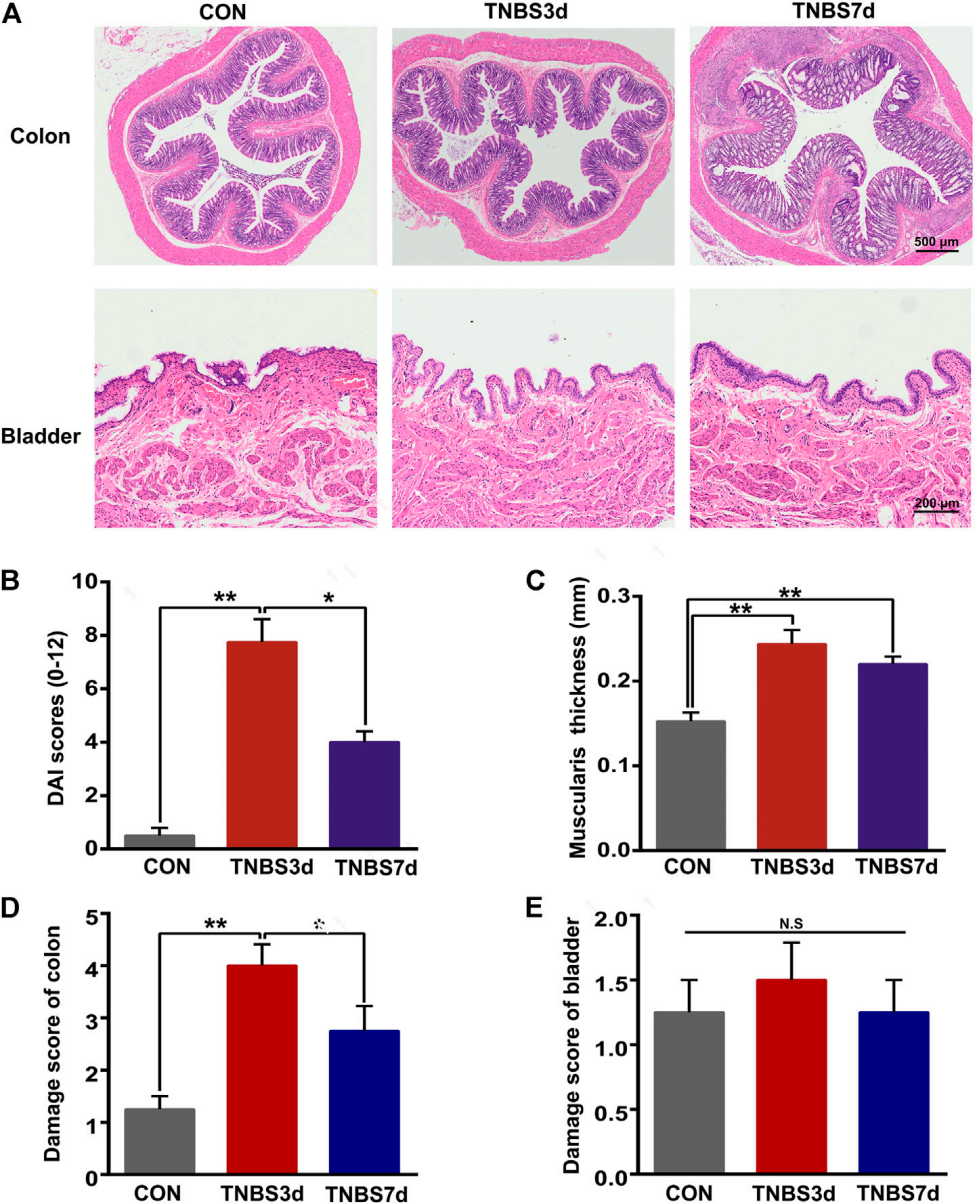
Histological changes of the distal colon and bladder in TNBS-induced colitis. **(A)** Representation of H&E staining of the distal colon (upper) and bladder (lower), and TNBS treatment led to inflammation of the distal colon but not the bladder. n = 4 for each group. **(B)** Histogram showed that the DAI score was significantly increased at day 3 after TNBS instillation, and the increased DAI score was partially recovered at day 7 post-TNBS. *p < 0.05 and **p < 0.01 by Kruskal–Wallis H-test. **(C)** Histogram showed that muscularis propria thickness was significantly increased at days 3 and 7 after TNBS instillation. **p < 0.01 by one-way ANOVA followed by Newman–Keuls post hoc tests. **(D)** Histogram showed the higher damage score of the distal colon after TNBS treatment, especially at day 3 post-TNBS. *p < 0.05, **p < 0.01 by Kruskal–Wallis H-test. **(E)** No significant differences in the damage score were detected in rat bladder among three groups by the Kruskal–Wallis H-test.

To evaluate the pathological changes in TNBS-induced colitis, the H&E staining of the distal colon and bladder was performed primarily. As shown in Figure 1A, the presence of mucosal hyperemia and edema, inflammatory cell infiltration, and the width of the submucosal spaces were observed at day 3 after TNBS instillation, verifying the development of acute colitis. Additionally, the DAI score and muscularis propria thickness were significantly increased at day 3 after TNBS instillation (Figures 1B,C, *p* < 0.01). By day 7, after TNBS instillation, the increased DAI score was recovered partially (Figure 1B, *p* < 0.05), but the muscularis propria thickness had no significant change (Figure 1C, *p* > 0.05), indicating the chronic inflammation of the distal colon. Moreover, the damage score of the colon at day 3 post-TNBS was significantly higher than that in control rats (Figure 1D, *p* < 0.01), and the damage degree at day 7 post-TNBS was partially alleviated (Figure 1D, *p* < 0.05). Interestingly, no significant histological changes were detected in rat bladder among groups, in terms of thickness of urothelium and suburothelium space and vascular density (Figure 1E, *p* > 0.05). These results suggested that TNBS-induced colitis has no effect on the histological change in the bladder.

### 3.2 TNBS-induced colitis increased P2X3 expression in the distal colon and bladder

**FIGURE 2:**
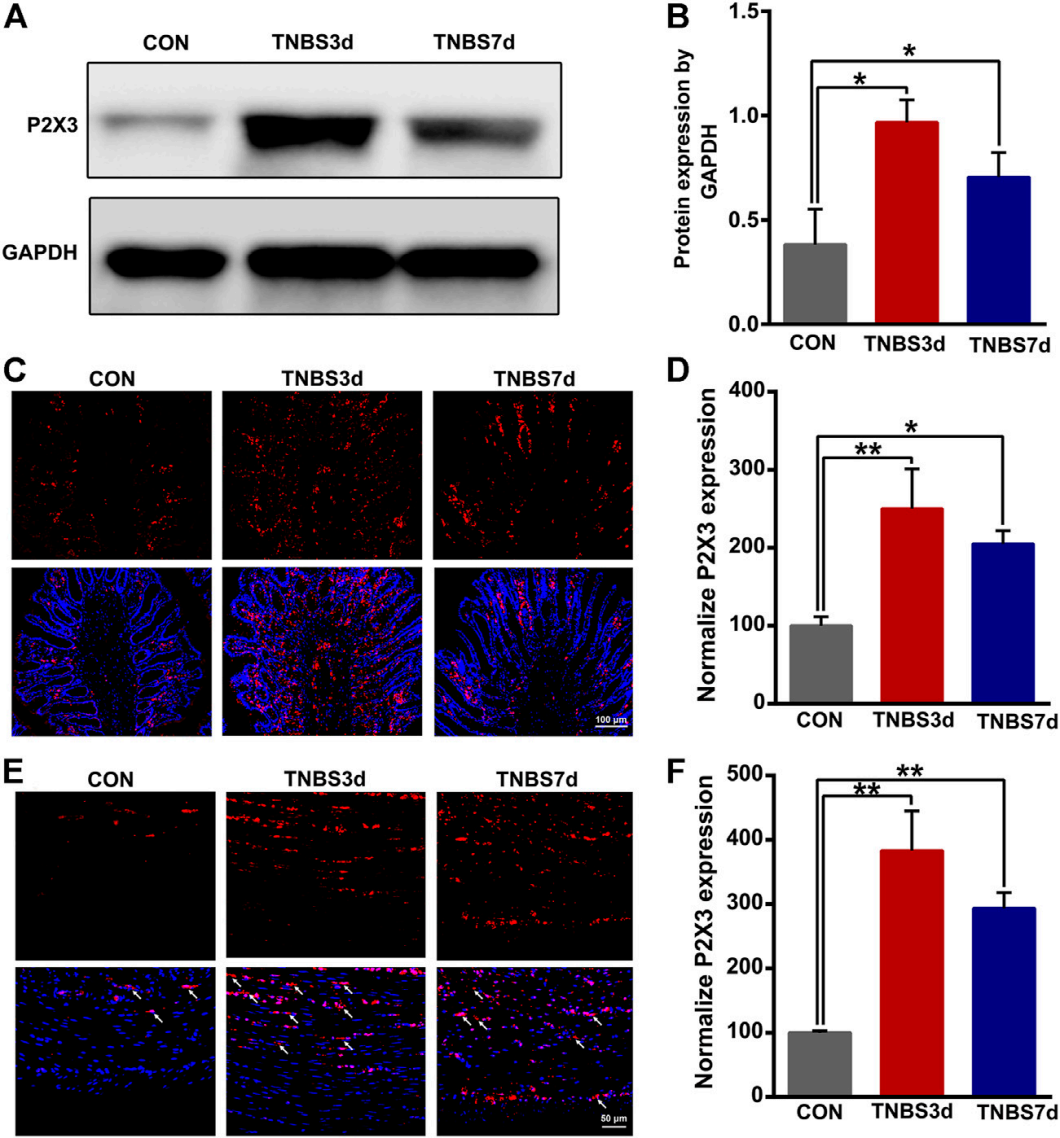
TNBS-induced colitis increased the P2X3 expression in the distal colon. **(A–B)** Western blotting showed that TNBS-induced colitis significantly increased the protein expression of the P2X3 receptor in the distal colon. n = 3 for each group. **(C–D)** P2X3 immunoreactivity in the submucosal plexus of the distal colon showed a significant increase in colitis at days 3 and 7 post-TNBS compared to the control group. Bar = 100 μm; n = 4 for each group. **(E–F)** P2X3 immunoreactivity in the muscular layer of the distal colon showed that TNBS-induced colitis significantly increased P2X3 expression compared to the control group. Bar = 50 μm; *n* = 4 for each group. *p < 0.05 and **p < 0.01 by one-way ANOVA followed by Newman–Keuls post hoc tests.

**FIGURE 3:**
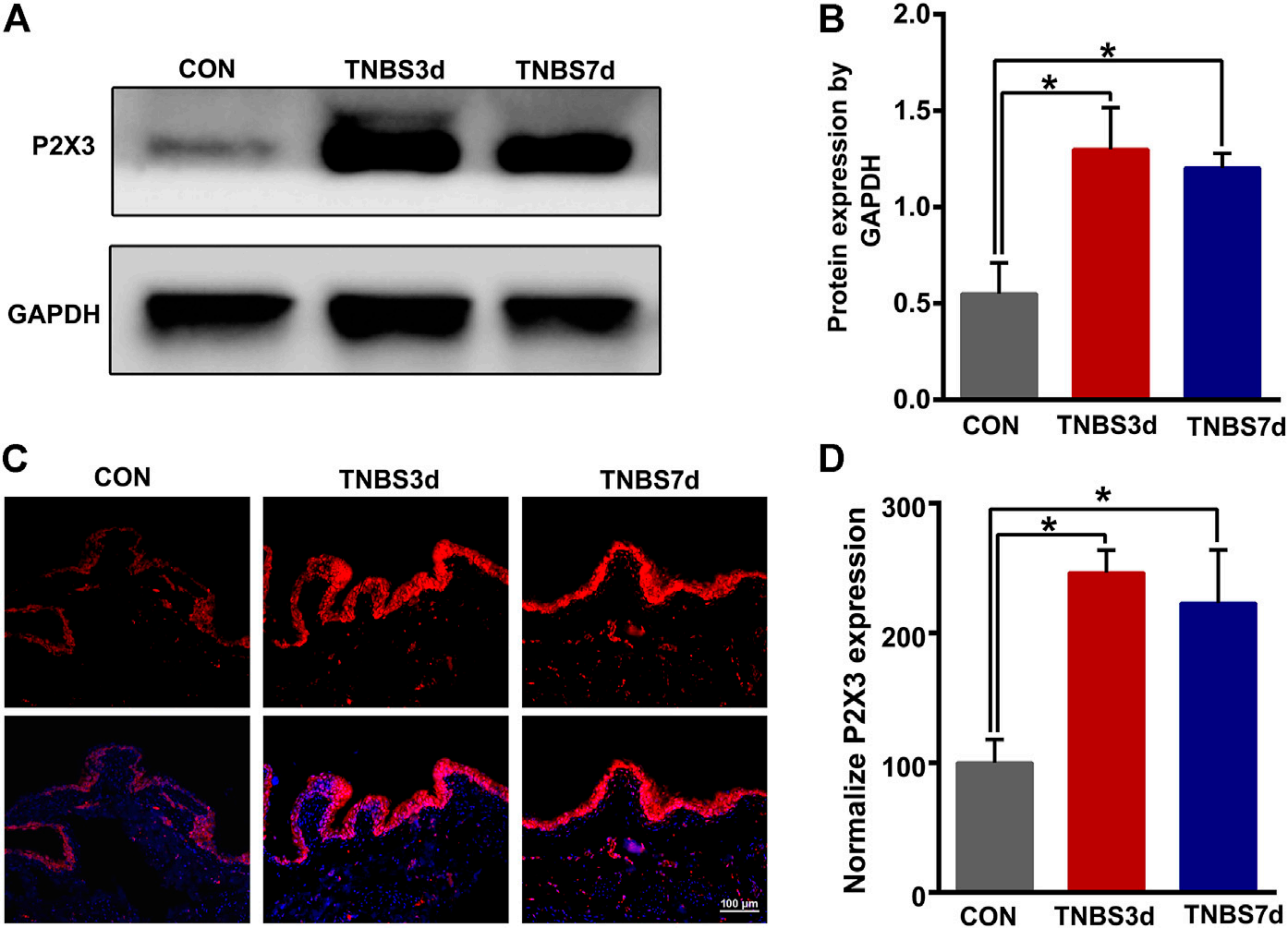
TNBS-induced colitis increased P2X3 expression in the bladder. **(A–B)** Western blotting showed that TNBS-induced colitis significantly increased the protein expression of the P2X3 receptor in the bladder. n = 3 for each group. **(C–D)** P2X3 immunoreactivity in the bladder showed that colitis significantly increased P2X3 expression of the urothelium of the bladder at days 3 and 7 post-TNBS compared to the control group. Bar = 100 μm; n = 4 for each group. *p < 0.05 by one-way ANOVA followed by Newman–Keuls post hoc tests.

To evaluate the expression of P2X3 receptors in the distal colon, we detected total P2X3 expression in the distal colon using Western blot. As shown in Figures 2A,B, P2X3 expression after TNBS treatment was significantly increased in the distal colon compared to the control group, especially at day 3 post-TNBS (Figures 2A,B, *p* < 0.05). The immunofluorescence staining of P2X3 antibody showed that P2X3 receptors were labeled on the myenteric and submucosal plexus of the distal colon (Figures 2C,E), which was consistent with the location of the nerve fiber in the distal colon. P2X3 expression of the submucosal plexus or muscular layer from TNBS-3-day groups was significantly higher than that of the control group (Figure 2D, *p* < 0.01, Figure 2F, *p* < 0.01). By day 7 after TNBS instillation, P2X3 expression was partially reduced but was significantly higher than that of the control group (Figure 2D, *p* < 0.05, Figure 2F, *p* < 0.01). Similarly, we found that total protein expression of P2X3 in the bladder at day 3 and day 7 post-TNBS was significantly increased in the bladder compared to that of the control group (Figures 3A,B, *p* < 0.05). Additionally, P2X3 receptors were mainly expressed on the urothelium of the bladder (Figure 3C). Treatment with TNBS also increased P2X3 expression in urothelium at days 3 and 7 after TNBS instillation compared to the control group (Figure 3D, *p* < 0.05). These results suggested that TNBS-induced colonic inflammation increased P2X3 expression in the distal colon and bladder.

### 3.3 TNBS-induced colitis increased P2X3 expression in DRG

**FIGURE 4:**
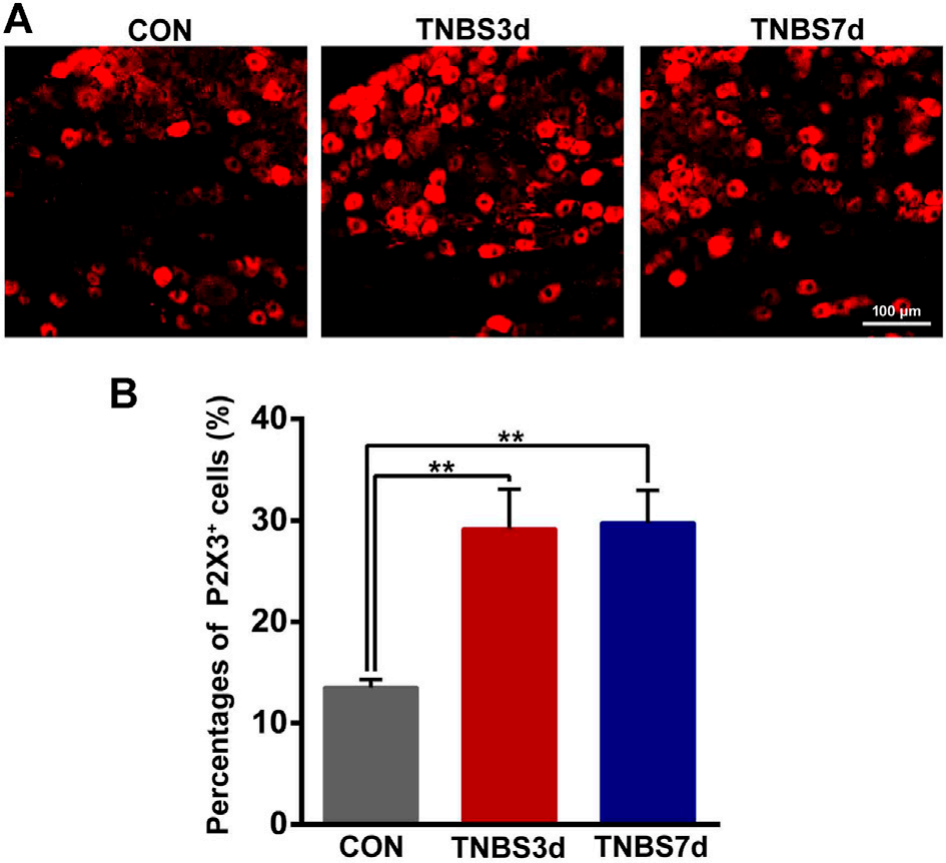
TNBS-induced colitis increased P2X3 expression in the neurons of L6-S1 DRG. **(A)** Immunofluorescence labeling of the P2X3 receptor in L6-S1 DRG showed that TNBS-induced colitis increased P2X3 expression on the small- and middle-sized neurons. **(B)** Histogram showed that the percentage of P2X3-positive neurons at days 3 and 7 post-TNBS was significantly higher than that of the control group. Bar = 100 μm; n = 4 for each group. **p < 0.01 by one-way ANOVA followed by Newman–Keuls post hoc tests.

Previous neuroanatomical tracing experiments have demonstrated that L6-S1 DRG contributed to bath distal colon and bladder sensory innervation (Herrity et al., 2014). To verify the effect of colonic inflammation on P2X3 expression of DRG neurons, we performed immunofluorescence staining of P2X3 antibody on L6-S1 DRG. As shown in Figure 4A, the P2X3 receptors were expressed on the small- and middle-sized neurons, primarily conducting the nociceptive and chemical stimuli. Moreover, treatment with TNBS instillation significantly increased the P2X3 expression in DRG neurons (Figure 4A). The percentage of P2X3 positive neurons in DRG was confirmed by an about two-fold increase at days 3 and 7 post-TNBS treatment compared to control groups (Figure 4B, *p* < 0.01). These results suggested that TNBS-induced colonic inflammation increased P2X3 expression in DRG neurons, possibly resulting in the increase of P2X3 expression in the bladder afferent pathway.

### 3.4 The inhibition of P2X3 alleviated bladder overactivity but not detrusor overactivity evoked by colitis

**FIGURE 5:**
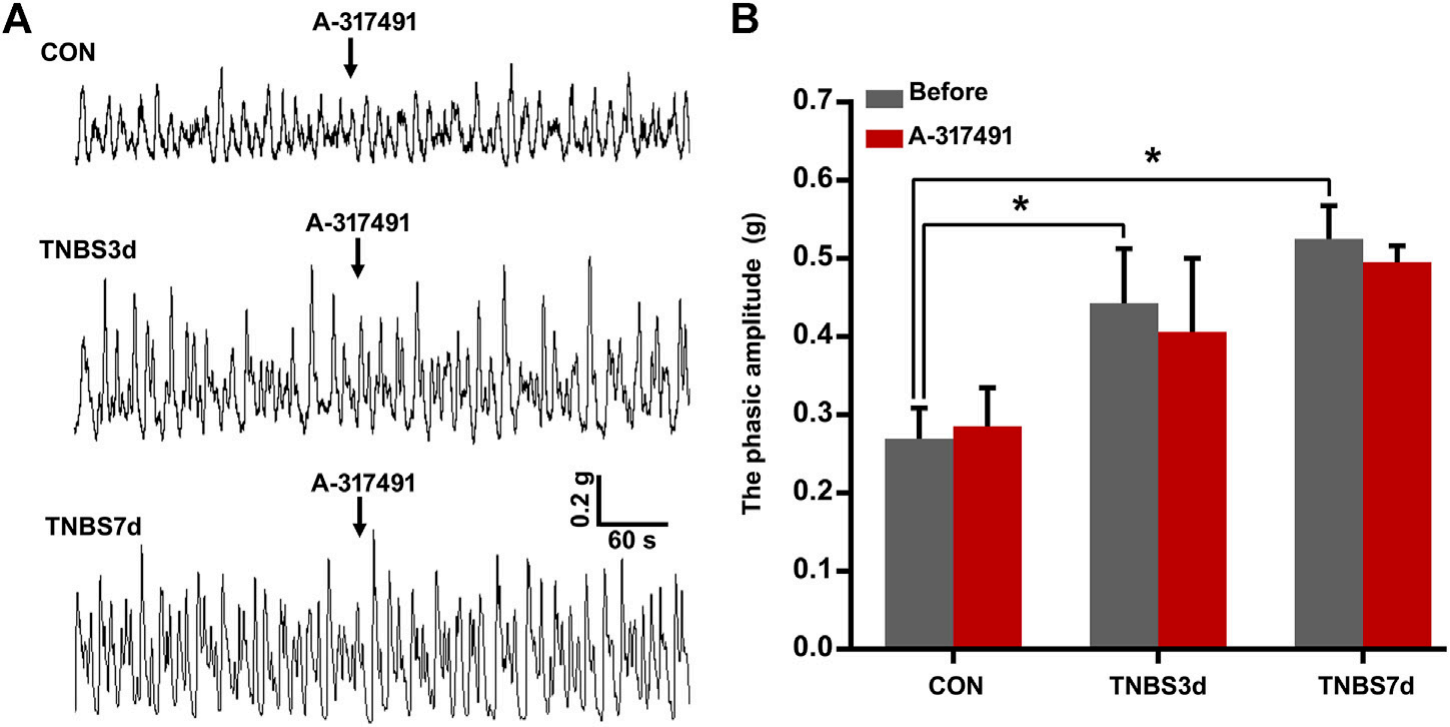
Spontaneous contractions of the detrusor strip isolated from the bladder of three groups had no sensitivity to the inhibition of P2X3 receptors. **(A)** Representative tracers of detrusor strip contractility responded to A-317491. The detrusor strip isolated from colitis rats exhibited the characteristic of detrusor overactivity. *n* = 4 for each group. **(B)** Histogram showed that TNBS-induced colitis significantly increased the phasic amplitude of detrusor strip contractility compared to the control group, but treatment with A-317491 had no effect on the detrusor strip contractility isolated from both control and colitis groups. *p < 0.05 by one-way ANOVA followed by Newman–Keuls post hoc tests.

It has been demonstrated that detrusor overactivity plays an important role in the colon–bladder cross-sensitization (Fitzgerald et al., 2013). To further investigate the function of P2X3 in the detrusor overactivity, the detrusor muscle spontaneous contractility was measured using isolated bladder strips. As shown in Figure 5, the spontaneous contractility of detrusor muscle in TNBS-treated rats appeared as unstable contraction and overactivity. The average phasic amplitude of spontaneous contractions at days 3 and 7 after TNBS instillation was significantly increased compared to the control group (Figures 5A,B, *p* < 0.05). However, A-317491, a P2X3 selective antagonist, was not found significantly effective on the detrusor overactivity at 3 and 7 days post-TNBS treatment (Figures 5A,B, *p* > 0.05). These results suggested that the upregulation of P2X3 receptors had no relation with colonic inflammation-induced detrusor overactivity, implying further that P2X3 receptors were involved in bladder overactivity by the primary afferent pathway.

**FIGURE 6:**
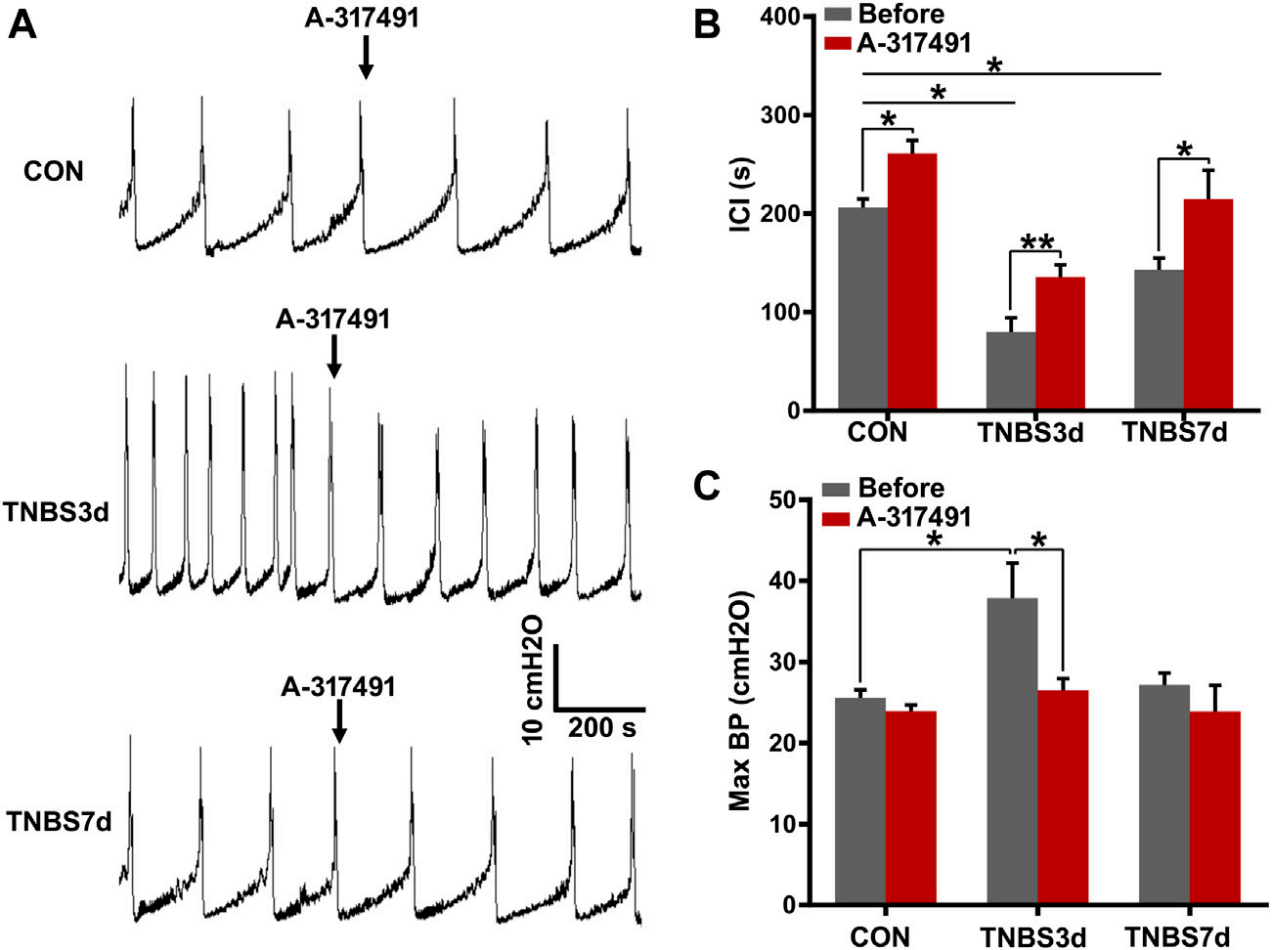
Inhibition of P2X3 alleviated bladder overactivity evoked by TNBS-induced colitis. **(A)** Representative tracers of cystometry patterns from three groups responded to A-317491 by intrathecal injection. The rats with colitis exhibited bladder overactivity, manifested by increased micturition frequency and bladder pressure. *n* = 4 for each group. **(B)** Histogram showed that the ICI of rats treated with TNBS was significantly shorter than that of the control group, and intrathecal injection with A-317491 significantly prolonged the ICI of both control and colitis groups. **(C)** Histogram showed that colitis significantly increased the maximum BP of rats at day 3 post-TNBS but not at day 7 post-TNBS. Intrathecal injection with A-317491 significantly inhibited the increased maximum BP at day 3 post-TNBS. *p < 0.05, **p < 0.01 by Student’s t-test or one-way ANOVA followed by Newman–Keuls post hoc tests.

To verify our speculation, the cystometry was performed on unconscious rats, and A-317491 was locally applied by intrathecal injection. The results at day 3 post-TNBS showed that the intercontractile interval was significantly prolonged (Figures 6A,B, *p* < 0.05), and maximum bladder pressure was enhanced (Figure 6C, *p* < 0.05). As chronic inflammation developed at day 7 post-TNBS, the intercontractile interval was still significantly shorter than that in control groups (Figures 6A,B, *p* < 0.05), but maximum bladder pressure had no significant difference from the control group (Figure 6C, *p* > 0.05). Inversely, A-317491 significantly inhibited the decrease in the intercontractile interval (Figures 6A,B, *p* < 0.01) and increase in maximum bladder pressure (Figure 6C, *p* < 0.05) at day 3 post-TNBS. Similarly, the decrease in the intercontractile interval at day 7 post-TNBS was reversed to the normal level (Figure 6B, *p* > 0.05). These results demonstrated that the P2X3 receptor was involved in bladder overactivity by the primary afferent pathway, and inhibiting the P2X3 receptor could alleviate the colonic inflammation-induced bladder overactivity.

## 4 Discussion

In this study, we established the rat models of colitis induced by intracolonic TNBS administration. The TNBS-induced colitis significantly increased the expression of P2X3 receptors on the myenteric and submucosal plexus of the distal colon and the urothelium of the bladder, especially 3 days after TNBS administration. Meanwhile, the expression of P2X3 receptors on DRG neurons was increased at TNBS-induced colitis. The rats with colitis displayed the defining characteristics of detrusor overactivity and bladder overactivity. Interestingly, inhibition of the P2X3 receptor alleviated bladder overactivity but showed an effect on detrusor overactivity. These results suggested that the upregulation of P2X3 receptors in afferent pathways involved in bladder overactivity evoked by TNBS-induced colitis.

An increasing number of animal studies give support for a clinical investigation that colonic inflammation has a close correlation with bladder overactivity of unknown etiology, such as IC/PBS (Cory et al., 2012; Bullones Rodriguez et al., 2013). In animal models of colitis, the changes in bladder voiding parameters include the decrease in micturition intervals, bladder capacity, and voided volumes (Xia et al., 2012; Lei et al., 2013). In this study, we found that rats with colitis displayed the defining characteristics of detrusor overactivity and bladder overactivity without histological changes in the bladder. In contrast with the maximum bladder pressure at day 7 after TNBS administration, the increase in maximum bladder pressure at day 3 post-TNBS maybe on account of the direct inflammatory irritation on the bladder during severe inflammation of the distal colon (Grundy and Brierley, 2018). By day 7 after TNBS administration, the colonic inflammation spontaneously started recovering, and the increase in maximum bladder pressure recovered to a normal level. Interestingly, the increase in micturition frequency still existed at 7 or 10 days post-TNBS (Ustinova et al., 2007) and even persisted for a longer term after TNBS administration when there was no observable change of colonic histological morphology between the TNBS-treated and normal mice (Malykhina et al., 2013; Grundy et al., 2018b). These results demonstrated that transient colonic inflammation results in long-term effects on micturition function of the bladder, following the recovery of initial inflammatory irritation.

The cross-sensitization of the afferent pathway is considered a vital mechanism underlying functional bladder and gastrointestinal diseases, such as IC/PBS, IBS, and chronic pelvic pain (Qin et al., 2005; Cory et al., 2012). Our and other previous reports have verified that the sensitization of bladder afferent pathways involves bladder overactivity in IC/PBS (Grundy et al., 2018a; Yang et al., 2021). Consistent with our findings in TNBS-induced colitis, the colitis in rodent models can cause the occurrence of bladder overactivity due to the reason of cross-sensitization in primary afferent pathways (Xia et al., 2012). Colonic inflammatory irritation enhances the firing rates of bladder C-fibers and contributes to mechanical hypersensitivity in response to normal bladder distension (Ustinova et al., 2006; Ustinova et al., 2007). Similarly, the TNBS-induced colonic inflammation also leads to hyperexcitability of DRG neurons innervating the bladder, manifested by a lower threshold for action potential (Beyak et al., 2004; Malykhina et al., 2006; Lei and Malykhina, 2012). Specifically, retrograde-tracing studies have revealed that afferent nerve fibers innervating the bladder and colon are partially overlapped at the level of dorsal root ganglia (DRG) neurons and second-order neurons of the spinal cord (Qin et al., 2005; Christianson et al., 2007; Herrity et al., 2014). Therefore, the sensitization of colonic afferent induced by inflammatory irritation may contribute to cross-sensitization of bladder afferent pathways (Malykhina et al., 2004; Qiao and Grider, 2007). It has been reported that colonic inflammation can affect some molecular expression and function of DRG neurons innervating the bladder, including calcitonin gene-related peptide (CGRP), substance P, TRPV1, and brain-derived neurotrophic factor (BDNF) (Qiao and Grider, 2007; Pan et al., 2010; Xia et al., 2012).

ATP has been demonstrated as a crucial neurotransmitter, involving visceral sensory conduction by activating heteromultimers P2X2/3 receptors or heteromultimers P2X3 receptors (Burnstock, 2001), especially the P2X3 receptor, predominantly expressed on the small-to-medium diameter DRG neurons, is widely deemed to play an important role in visceral pain and hypersensitivity (Ford, 2012). Under physiological or pathological conditions, ATP is released from epithelial cells in response to distension of hollow organs (e.g., bladder, gut, and lung) and then activates the P2X3 receptor of peripheral afferent terminals to conduct the chemical and nociceptive sensory (Burnstock, 2001). Moreover, growing evidence has demonstrated that overactivation of the P2X3 receptor is closely relevant to visceral hypersensitivity of colitis (Wynn et al., 2004; Deiteren et al., 2015). In this study, we found that the colitis-induced increase in P2X3 receptors primarily existed in the submucosal plexus and myenteric plexus of the distal colon, which were consistent with the location of colonic afferent fibers (Xiang and Burnstock, 2004). Although several evidence have demonstrated that peripheral P2X3 receptors have no effect on colonic mechanosensitivity under physiological conditions but participate in TNBS-induced visceral hypersensitivity (Shinoda et al., 2009; Deiteren et al., 2015). The upregulation of the P2X3 receptor has been observed in DRG neurons from a rat model of colitis induced by TNBS (Wynn et al., 2004). Moreover, the P2X3 antagonist alleviates the increase in visceromotor response during colorectal distention in acute and post-inflammatory phases (Deiteren et al., 2015).

Growing evidence has demonstrated that the P2X3 receptor participates in micturition reflex and bladder nociception (Cockayne et al., 2000; Vlaskovska et al., 2001; Andersson, 2002). For example, the knockout of the P2X3 receptor in mouse shows bladder hyporeflexia, manifested by attenuated voiding frequency, but normal bladder pressure (Cockayne et al., 2000). Additionally, the activity of bladder afferent fibers in knockout mice exhibits attenuated responses to mechanical stimulation induced by gradual bladder distension (Vlaskovska et al., 2001). Similarly, we found that pharmacological inhibition of the P2X3 receptor prolonged the voiding frequency but had no effect on the bladder pressure and detrusor contraction. This evidence further verifies the conclusion that the P2X3 receptor participates in micturition reflex via the sensory afferent pathway, not through the efferent pathway or detrusor activity. Moreover, the upregulation of P2X3 receptors in the bladder afferent pathway has been demonstrated to be closely associated with pathological bladder overactivity, such as neurogenic bladder or cystitis (Brady et al., 2004; Dang et al., 2008). Pharmacological inhibition or knockout of the P2X3 receptor alleviates the bladder overactivity evoked by cyclophosphamide-induced cystitis (Ito et al., 2008). Consistent with cystitis (Dang et al., 2008), we found that the expression of the P2X3 receptor was increased in the urothelium of the bladder and DRG neurons. Inhibition of the P2X3 receptor significantly alleviated the bladder overactivity evoked by TNBS-induced colitis. This evidence suggested that the P2X3 receptor antagonist may be an available and novel strategy for the control of bladder overactivity.

## Data Availability

The original contributions presented in the study are included in the article/Supplementary Material; further inquiries can be directed to the corresponding authors.
